# Two Decades of Gastrointestinal Stromal Tumor Management With First-Line Treatment: A Case Report

**DOI:** 10.7759/cureus.71299

**Published:** 2024-10-12

**Authors:** Maria M Pereira, Elisabete Couto, Ali Shamseddine, Teresa Macedo

**Affiliations:** 1 Medical Oncology, Unidade Local de Saúde de Braga, Braga, PRT; 2 School of Medicine, Universidade do Minho, Braga, PRT; 3 Hematology/Oncology Division, American University of Beirut Medical Center, Beirut, LBN; 4 Paliative Care, Unidade Local de Saúde de Braga, Braga, PRT

**Keywords:** gastrointestinal stromal tumor (gist), imatinib and sunitinib, imatinib therapy, jejunal gist, mesenchymal tumors

## Abstract

Gastrointestinal stromal tumors (GISTs) are rare malignant tumors that arise from the connective tissues of the gastrointestinal tract. Reports about GISTs treated with imatinib for over five years are exceedingly rare. In this case report, we present a patient with GIST who remained alive for two decades after undergoing imatinib treatment. We describe a man in his forties with no significant prior medical issues, who presented in 2004 to the emergency department with severe abdominal pain. A computed tomography scan revealed a ruptured intra-abdominal mass. He underwent surgical resection, leading to the diagnosis of jejunal GIST. Following this, he underwent active surveillance until 2007, when the disease recurred. After completing a two-year course of imatinib, no signs of disease were detected, and medical therapy was stopped. However, in 2011, a pelvic recurrence was observed, leading to the re-introduction of imatinib therapy, which was maintained until 2018. A year later, disease progression was noted, prompting the re-initiation of imatinib. Since then, the patient was receiving imatinib therapy with good tolerance, until March of 2024. This case highlights the potential sensitivity of imatinib for a long duration.

## Introduction

Gastrointestinal stromal tumors (GISTs) are the most common sarcomas in the gastrointestinal tract. They are a rare malignant mesenchymal tumor whose incidence varies between 0.4 to two cases per 100,000 per year [1]. Surgical excision is the standard approach to tumors with more than 2 cm in size. Adjuvant therapy with imatinib, a tyrosine kinase inhibitor (TKI), for three years, is the standard treatment for patients with a high risk of relapse. Imatinib is also the standard treatment for metastatic patients, including patients previously treated with imatinib who did not relapse while receiving it [1,2]. Case reports about GISTs, treated with imatinib for more than five years, are extremely rare. Here, we report a case of gastrointestinal stromal tumor treated for two decades since the diagnosis. We present the following case in accordance with the CARE guidelines [3].

## Case presentation

**Figure 1:**
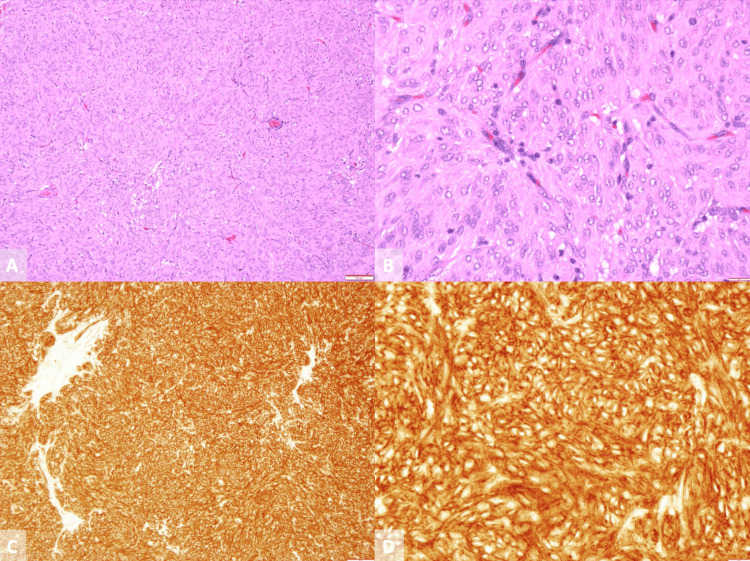
Pathological findings. (A) Hematoxylin-eosin staining, magnification 100x. (B) Hematoxylin-eosin staining, magnification 400x. (C) Immunohistochemical staining revealed that tumor cells were positive for CD177, magnification 100x. (D) Immunohistochemical staining, magnification 400x.

We report the case of a 49-year-old man with no relevant personal or family history. In 2004, he presented to the emergency department complaining of sudden onset of severe abdominal pain. Physical examination revealed abdominal tenderness in the epigastric region. Contrast-enhanced abdominal computed tomography (CT) was conducted and showed a ruptured intra-abdominal mass. The patient underwent surgical resection, and the histopathological analysis of the mass demonstrated spindle cells and five mitoses per 50 high-power fields (Figures 1A, 1B). Immunohistochemistry revealed a cluster of differentiation 117 (CD117) positivity, consistent with a diagnosis of jejunal gastrointestinal stromal tumor (Figures 1C, 1D).

**Figure 2:**
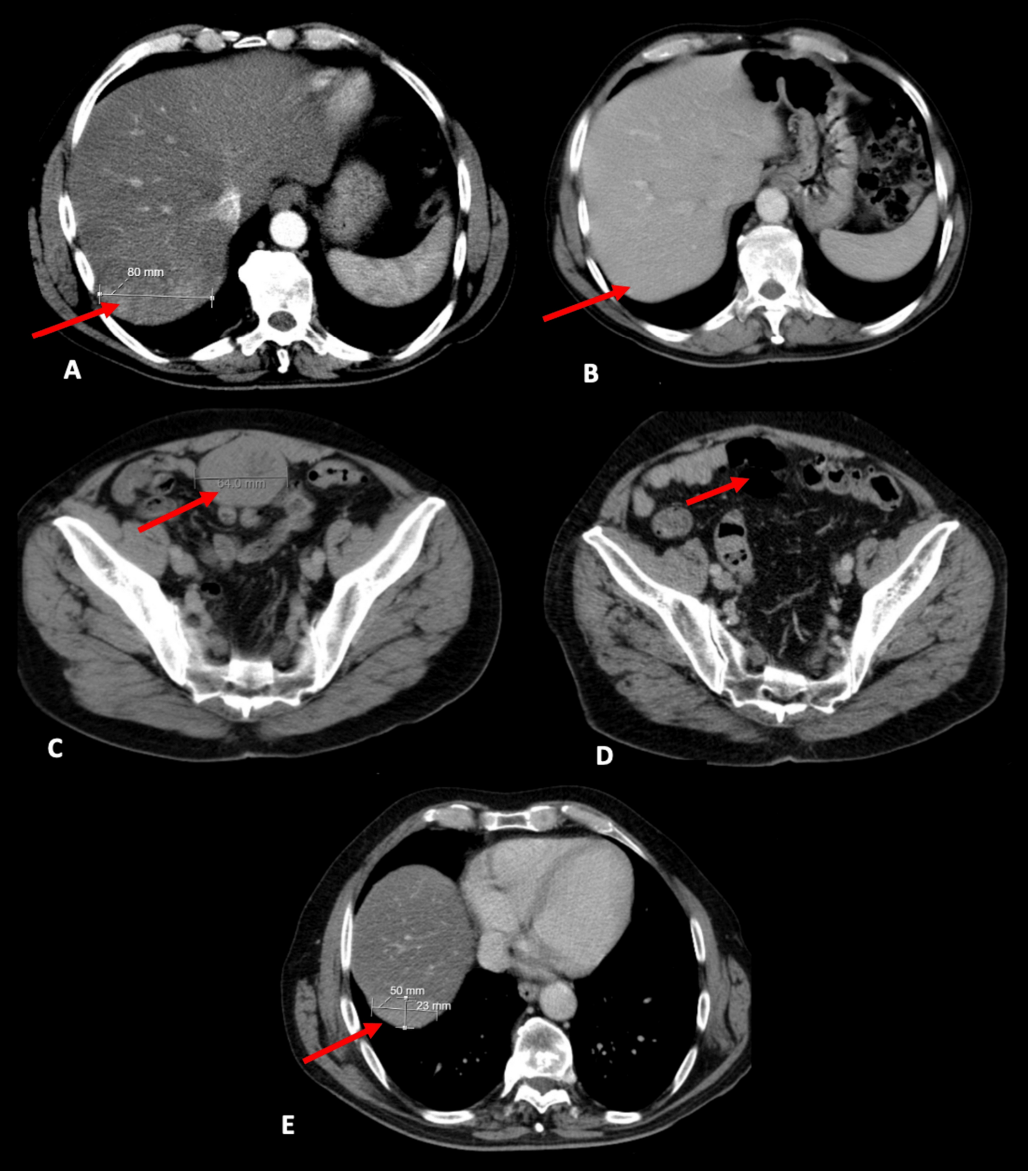
Computed tomography images of the gastrointestinal stromal tumor. (A) In 2007, prior to imatinib administration, a lesion emerged measuring 80 mm in the posterior right lobe of the liver. (B) By 2009, routine CT scans indicated the liver lesion had vanished. (C) In 2011, a novel pelvic lesion was observed. (D) By 2018, after six years of imatinib treatment, the pelvic mass had resolved. (E) In 2019, the disease recurred manifesting as a new lesion positioned above the diaphragm in proximity to the liver.

The patient remained in active surveillance until 2007, as imatinib as adjuvant therapy had not been approved at that time. In 2007, an enhanced CT scan showed a new lesion under the diaphragm, near segment VI of the liver (Figure 2A). The patient was asymptomatic and he was submitted to exploratory laparoscopy that showed peritoneal implants that were considered unresectable. The biopsy of the lesion was compatible with GIST metastasis. He was started on Imatinib 400mg once a day from November 2007 to November 2009. During treatment, a follow-up CT scan and positron emission tomography (PET) CT scan were done every three months, both of which showed no evidence of disease, indicating a complete response (Figure 2B).

The imatinib was stopped and the patient remained on surveillance until September 2011, when a new lesion in the anterior pelvic region appeared (Figure 2C). The resection of the single lesion confirmed that it was consistent with a metastasis of a GIST. Imatinib was restarted in February 2012 until 2018. In July 2018, due to grade 1 anemia and thrombocytopenia, the dose of imatinib was reduced to 200mg. Even after the dose reduction, the hematological toxicity persisted leading to the imatinib interruption. At that time, there was no evidence of disease (Figure 2D).

In 2019, the disease reappeared and imatinib was restarted (Figure 2E). Since that time, he has been receiving imatinib with adequate clinical control and there were no recurrences until his last check-up in March 2024, where the CT tomography revealed a new implant located in the right iliac fossa. The multidisciplinary tumor board decided to start second-line treatment with sunitinib. 

## Discussion

The primary management of GISTs is surgical, with complete surgical resection. However, after surgery, up to 50% of patients develop local recurrence or metastasis, mainly in the first three years [4]. Therefore, before the approval of TKIs as adjuvant therapy following GIST surgical resection, patients were managed by active surveillance only [5]. 

In 2002, imatinib was approved for unresectable and/or metastatic malignant GIST. Later, in 2008, data suggested that adjuvant treatment was associated with higher recurrence-free survival for patients with higher-risk disease [6]. It also has been shown that adjuvant treatment with imatinib after the surgery was the standard treatment, and 36 months of treatment was better than 12 months [6,7]. However, it was observed that upon discontinuance of imatinib, recurrence is very probable [8].

In the presented case, the patient had the first disease recurrence in 2007. The tumor rupture at the time of the diagnosis was an important risk factor predictive of recurrence, which left the patient with a very high risk of relapse [1].

The optimal duration of post-operative imatinib in patients with tumor rupture is not defined, given the uncertainty if these cases can be considered as already metastatic [9,10]. Even though, when the disease relapsed, as in 2012 and 2019, the patient restarted imatinib and the TKI was stopped when there wasn’t imagological evidence of the disease.

For this reason, nowadays, there is an understanding of the benefits of treatment with imatinib indefinitely in the metastatic setting. According to ESMO (European Society for Medical Oncology) guidelines, in metastatic disease, imatinib should be continued until clinically relevant disease progression or intolerance, because treatment interruption is generally followed by relatively rapid tumor progression [1]. 

Hence, we describe a case of gastrointestinal stromal tumor that has been treated with imatinib since 2007. There are a few reports about long-term treatment with imatinib in patients with GIST. One case report describes a patient who was treated with adjuvant therapy for twelve years without interruption until disease recurrence, which is different from our case because he had several breaks in the treatment [11]. Another report has some similarities with our case report, because the patient received adjuvant imatinib, however, two years after completion of medical therapy, local peritoneal recurrence of GIST was observed and medical therapy with imatinib was restarted. Despite the resumption of imatinib, the progression was observed one year after [12]. In our case progression with imatinib was only documented twenty years after the initial diagnosis, and this is the main reason for this publication. 

Moreover, some authors suggest that some patients can benefit from lifelong therapy and prolonged therapy, and this does not significantly increase treatment-related adverse effects. In this case, we can see that imatinib sensitivity can be maintained in a patient with multiple previous exposures to it with a good tolerance.

## Conclusions

This case report illustrates the long-term management and treatment outcomes of a patient with GIST treated with imatinib over a span of nearly two decades. The case highlights the complexity and challenges in treating GIST, particularly in the context of tumor recurrence and the need for ongoing therapy. Our patient experienced several recurrences, requiring the reinitiation of imatinib and dose adjustments due to hematological toxicity. Despite these challenges, the patient achieved significant periods of disease control, demonstrating the efficacy of imatinib in long-term management.

The report underscores the importance of individualized treatment plans and the role of a multidisciplinary approach in managing recurrent GIST. The extended use of imatinib, even after interruptions, proved effective in maintaining disease stability for this patient, aligning with current guidelines that recommend continuous treatment until disease progression or intolerance. This case contributes to the growing body of evidence supporting the potential benefits of lifelong imatinib therapy for certain patients with metastatic GIST.
